# Influence of femoral tunnel exit on the 3D graft bending angle in anterior cruciate ligament reconstruction

**DOI:** 10.1186/s40634-021-00364-9

**Published:** 2021-06-25

**Authors:** Sandro Hodel, Sylvano Mania, Lazaros Vlachopoulos, Philipp Fürnstahl, Sandro F. Fucentese

**Affiliations:** 1 Department of Orthopedics, University of Zurich, Balgrist University Hospital, Forchstrasse 340, 8008 Zurich, Switzerland; 2 Research in Orthopedic Computer Science (ROCS), University Hospital Balgrist, University of Zurich, Forchstrasse 340, 8008 Zurich, Switzerland

**Keywords:** ACL, Anterior cruciate ligament, Graft bending angle, GBA, Femoral tunnel

## Abstract

**Purpose:**

To quantify the influence of the femoral tunnel exit (FTE) on the graft bending angle (GBA) and GBA-excursion throughout a full range of motion (ROM) in single-bundle anterior cruciate ligament (ACL) reconstruction.

**Methods:**

Three-dimensional (3D) surface models of five healthy knees were generated from a weight-bearing CT obtained throughout a full ROM (0, 30, 60, 90, 120°) and femoral and tibial ACL insertions were computed. The FTE was simulated for 16 predefined positions, referenced to the Blumensaat's line, for each patient throughout a full ROM (0, 30, 60, 90, 120°) resulting in a total of 400 simulations. 3D GBA was calculated between the 3D directional vector of the ACL and the femoral tunnel, while the intra-articular ACL insertions remained unchanged. For each simulation the 3D GBA, GBA-excursion, tunnel length and posterior tunnel blow-out were analysed.

**Results:**

Overall, mean GBA decreased with increasing knee flexion for each FTE (*p *< 0.001). A more distal location of the FTE along the Blumensaat's line resulted in an increase of GBA and GBA-excursion of 8.5 ± 0.6° and 17.6 ± 1.1° /cm respectively (*p *< 0.001), while a more anterior location resulted in a change of GBA and GBA-excursion of -2.3 ± 0.6° /cm (+ 0.6 ± 0.4°/ cm from 0–60° flexion) and 9.8 ± 1.1 /cm respectively (*p *< 0.001).

Mean tunnel length was 38.5 ± 5.2 mm (range 29.6–50.5). Posterior tunnel blow-out did not occur for any FTE.

**Conclusion:**

Aiming for a more proximal and posterior FTE, with respect to Blumensaat’s line, reliably reduces GBA and GBA-excursion, while preserving adequate tunnel length. This might aid to reduce excessive graft stress at the femoral tunnel aperture, decrease femoral tunnel widening and promote graft-healing.

**Level of Evidence:**

IV

## Introduction

Anterior cruciate ligament (ACL) reconstruction is widely used to restore knee function and successful graft incorporation is key to restore knee stability and prevent graft failure. Numerous factors have been shown to influence graft failure after ACL reconstruction, as biological [21], laxity [35], graft choice [11], fixation technique [10], and anatomical tunnel positioning [25].

Previous studies have recently supported the role of the graft bending angle (GBA) in graft incorporation as more acute GBAs might lead to excessive stress between the graft and the anterior femoral tunnel aperture [1, 7]. From a biomechanical point of view, not only the GBA but also the GBA-excursion throughout the full knee range of motion (ROM) is likely to play an important role in graft stress and friction at the femoral tunnel inlet. This effect potentially impairs essential bone to tendon healing and is associated with increased femoral tunnel widening [17] and possibly explains the higher graft failure rate at the location of the femoral tunnel aperture [20]. Decreased graft maturation in the presence of an acute GBA has been confirmed with the signal/noise quotient (SNQ) in magnetic resonance imaging (MRI), especially in the early phase of graft incorporation [13, 16]. Moreover, GBA is known to be accentuated during motion and weight bearing activities and increasing stress on the proximal bone-graft interface [31]. Another factor to be considered regarding the femoral tunnel is its length [32], which contributes to initial fixation strength [6, 34].

The role of the femoral tunnel exit (FTE) has only been studied scarcely. For example, previous studies reported a more acute GBA with a more anterior FTE with the use of flexible drills or depending on the technique (outside in vs. transportal vs. transtibial) [14, 30]. However, no systematic analysis of the FTE and its relationship with GBA, GBA-excursion and femoral tunnel length throughout a full range of motion under weight-bearing conditions exists to our knowledge. We hypothesized that the location of the FTE significantly influences the GBA. Therefore, the aim of the study was to quantify the influence of the FTE on GBA, GBA-excursion and femoral tunnel length in single-bundle ACL reconstruction under weight-bearing conditions.

## Methods

After local review board approval, weight-bearing computer-tomography (CT) scans of five male volunteers with a mean age of 36 years (range 29 to 42 years), obtained for a previous study [5] were used for the 3D simulation. No participant had previous knee injury or surgery. High-resolution CT images of the left knee in increasing knee flexion (0, 30, 60, and 120°) were acquired using an open extremity CT scanner (Verity, Planmed, Norway ©; slice thickness 0.4 mm). All scanned knees were included for analysis.

3D triangular surface models were computed with manual threshold segmentation and region growing using MIMICS software (MIMICS, Materialize, Belgium). Afterwards, the models were imported into the in house developed planning software CASPA (Balgrist, Zurich, Switzerland). The femur remained stationary at 0° flexion as a reference and the tibia motion was defined relative to the femur during flexion. The femur models of each subject were superimposed using an iterative closest point surface registration algorithm [3] and five increasing knee flexion angles (0, 30, 60, 90, 120°) were interpolated to minimize the effect of different degrees of flexion among individuals from data acquisition.

### Definition of intra-articular ACL insertions

The intra-articular tibial and femoral ACL insertion points were defined based on weighted means of anatomic insertion sites as described by Parkar et al. [24] who reported the tibial insertions according to Stäubli et al. [29] and the femoral insertions according to Bernard et al. [2].

**Fig. 1:**
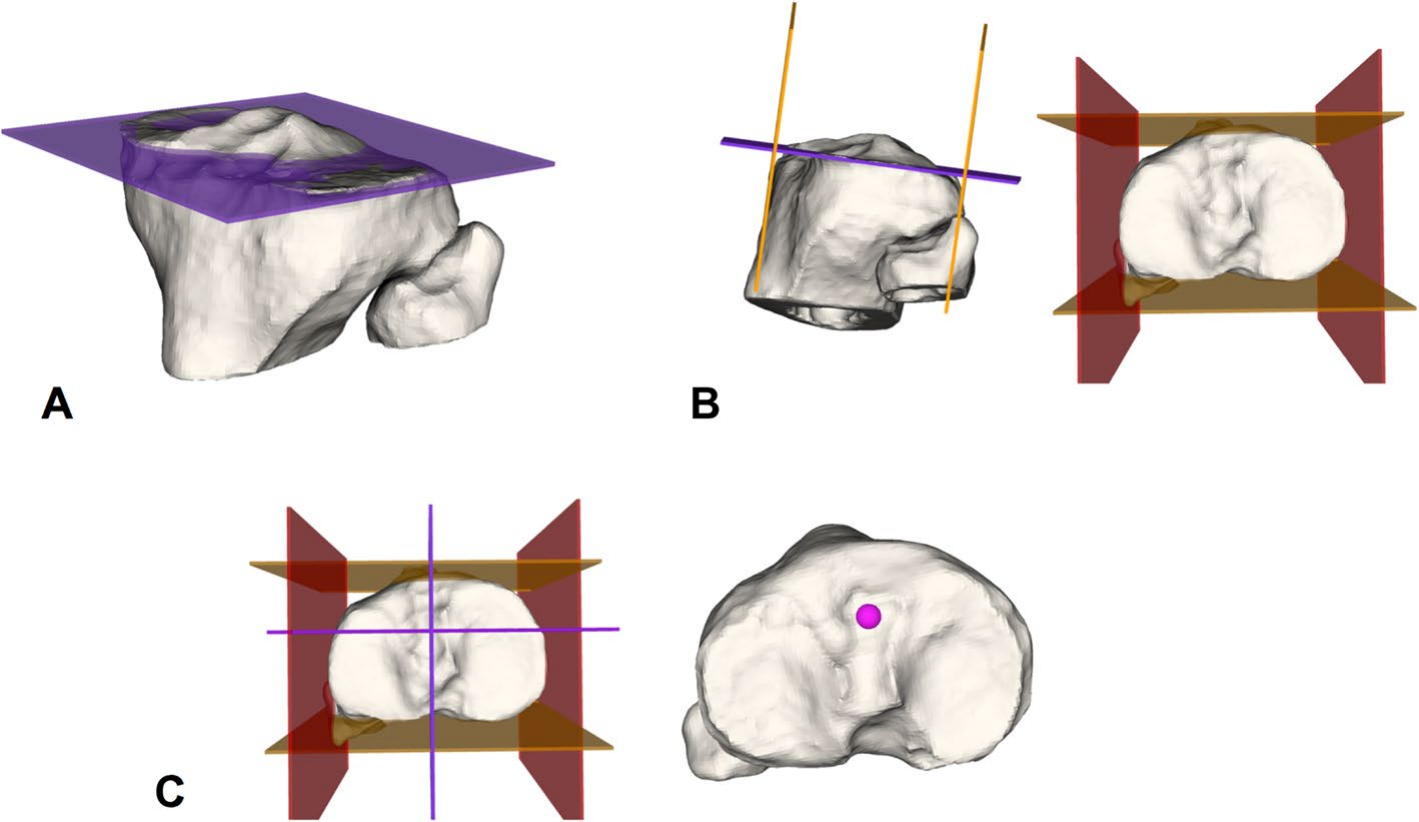
Tibial ACL insertion. Definition of 3D tibial ACL insertion based on Stäubli et al. [29] and described in the text

The tibial ACL insertion points were defined as follows (Fig. 1):

1. A plane was fitted to the tibial joint plane by defining three surface points on the medial and lateral plateau in a standardized way (A).
2. Anterior and posterior border planes (orange) were defined to be tangent to the most anterior and posterior margin of the tibial plateau with the normal vector being the cross product of the normal vector of the tibia joint plane and the tangent vector to the posterior condyles. Analogue a medial and lateral border plane (red) were defined being perpendicular to the anterior/posterior boarder plane and the tibial joint plane (B).
3. The anterior border was shifted 42.3% of the total antero-posterior distance posteriorly and the medial border plane was shifted to 50% of the mediolateral distance to the middle (violet intersection). The resulting intersection with the tibial plateau defined the ACL tibial insertion (pink) (C).

**Fig. 2:**
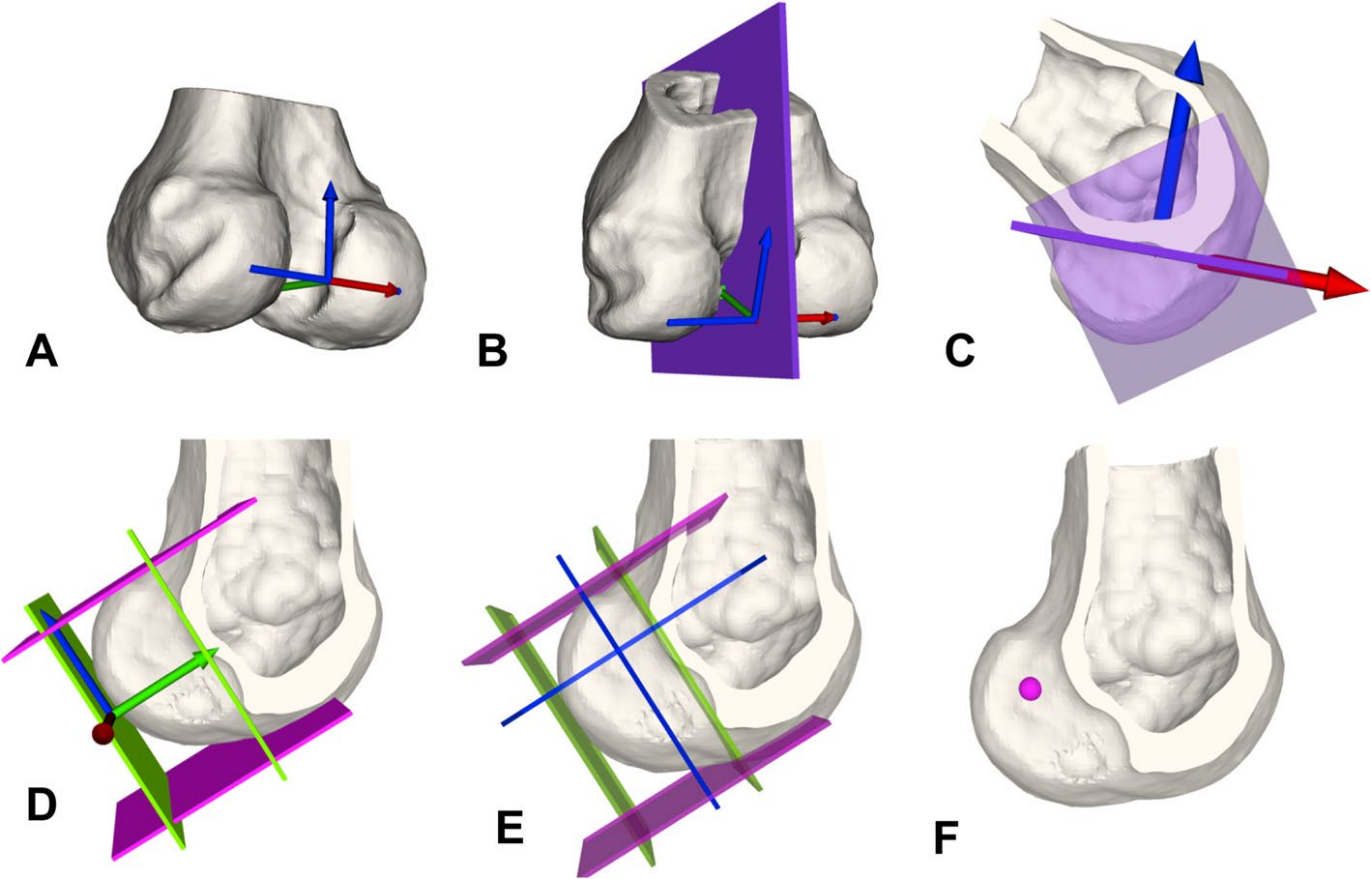
Femoral ACL insertion. Definition of 3D femoral ACL insertion based on Bernard et al. [2] and described in the text

The femoral ACL insertion was simulated as follows (Fig. 2):

1. The vector between the most prominent points on the posterior medial and lateral femur condyle was defined as the posterior condyle line (A).
2. A perpendicular sagittal cut plane (violet) to the posterior condyle line was created through the center of the posterior condyle line to visualize the Blumensaat’s line (B, C).
3. High and low border planes (green) were defined to be tangent to the Blumensaat’s line and posterior margin of the femoral condyle with the normal vector being the cross product of the normal vector of the sagittal cut plane and the tangent vector to the Blumensaat’s line. Analogue a deep and shallow border plane (pink) were defined being perpendicular to the anterior/posterior boarder plane and the sagittal cut plane (B).
4. The *deep* plane was shifted 28.6% in direction shallow and the high plane was shifted 34.5% in direction *low* (blue intersection) (E). The resulting intersection with the femur defined the femoral ACL insertion site (F).

**Fig. 3:**
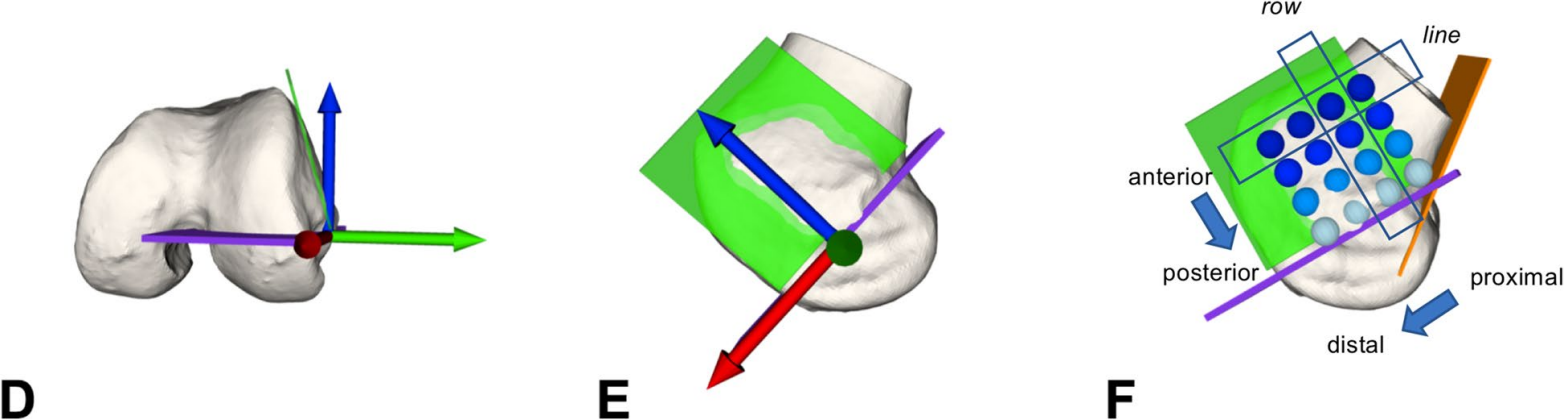
Femoral tunnel exit (FTE). Definition of femoral tunnel exit and orientation of rows (proximal to distal) and lines (anterior to posterior)

The FTE points were defined as follows (Fig. 3):

1. Steps A-C are equal to Fig. 2.
2. Blumensaat's plane was duplicated and rotated around the x-axis (red arrow) of its own coordinate system until fitting to the inclined lateral cortical surface and referred to as the lateral cortical plane (green) (D, E).
3. Additionally, to define the most posterior aspect of the FTE a plane tangent to the posterior cortical surface was created and referred to as the posterior cortical plane (orange). First, the most proximal and posterior point was defined without violating the intersection of the Blumensaat’s plane (violet) and the posterior cortical plane (orange) to avoid tunnel blow-out. Subsequently, 16 spheres (8 mm diameter) with a distance of one cm between centers were created with their centers intersecting the lateral cortical plane. Referenced to the Blumensaat's plane, four points forming a *row* and the direction to the posterior cortical plane is subsequently referred to as proximal and the opposite distal. Four points forming a *line* and the points closer to the Blumensaat's plane are referred to as posterior and the opposite anterior (F).

### Graft bending angle (GBA), GBA-excursion and tunnel length

While the intra-articular insertion was left unchanged, the FTE was simulated on the lateral cortical surface of the femur at 16 predefined points (Fig. 3) for each knee flexion position (0, 30, 60, 90, 120°) resulting in 400 simulations. The femoral tunnel diameter was set to 8 mm, representing an average tunnel size.

**Fig. 4:**
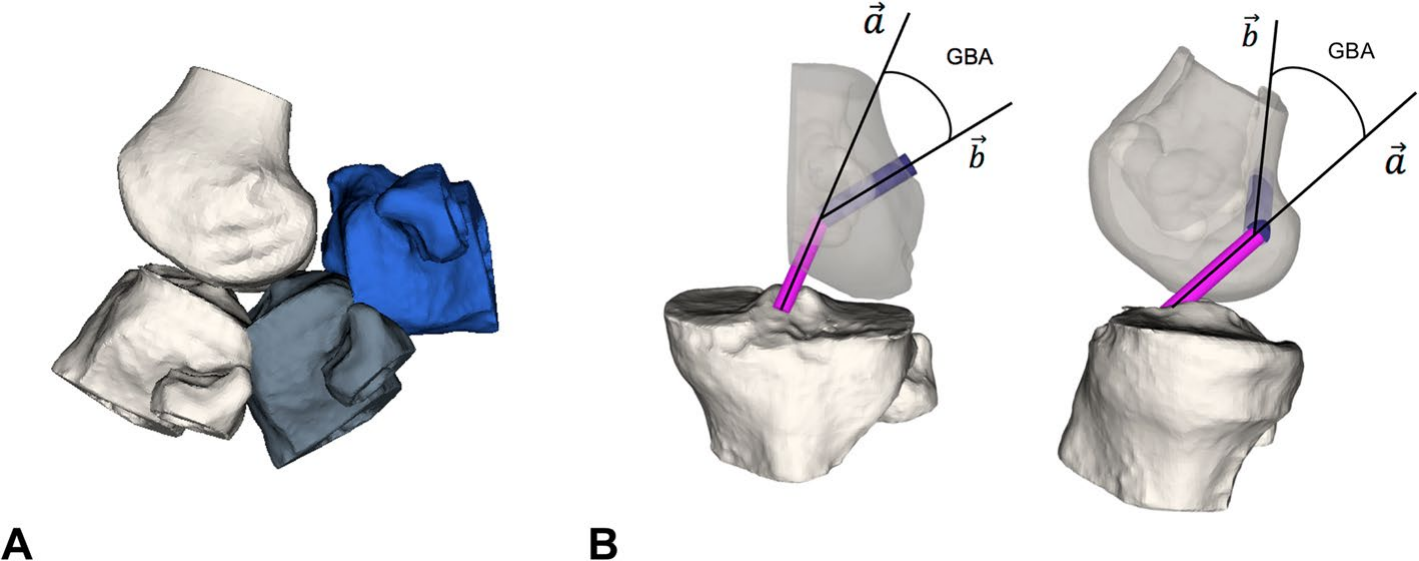
Schematic illustration of different flexion grades and measurement of graft bending angle (GBA). GBA: Graft bending angle. The tibia moved around the femur for 0, 30, 60, 90, 120° of flexion (**A**). 3D GBA between ACL (pink) and femoral tunnel (violet) illustrated from an anterior and lateral view (**B**)

The 3D GBA (α) was calculated using the scalar product of the 3D directional vector of the ACL ($\overrightarrow{a}$) and the 3D directional vector of the simulated femoral tunnel ($\overrightarrow{b}$). The ACL vector was defined from the tibial to the femoral insertion point and the femoral tunnel vector between the femoral insertion site and the simulated FTE (Fig. 4):

$$\alpha ={\mathrm{cos}}^{-1}\left(\frac{\overrightarrow{a}*\overrightarrow{b}}{\left|\overrightarrow{a}\right|\bullet \left|\overrightarrow{b}\right|}\right)$$

The GBA-excursion was defined as follows: *ΔGBA* = *GBA (0° flexion) – GBA (120° flexion).*

Additionally, for each simulation the tunnel length and posterior blow-out were evaluated. Tunnel length was defined from the center of the femoral ACL insertion to the center of the FTE and corrected for the radius of the femoral exit sphere (4 mm), as the spheres were projected to the surface of the femur corticalis. To correct for absolute body height, tunnel length was adjusted to the distance from the medial to the lateral epicondyle as follows: *Tunnel length corr* = Absolute tunnel length (mm) * epicondylar distance (mm)/100. Posterior blow out was defined if the femoral tunnel breached the posterior femur corticalis.

### Statistics

Normal distribution of the data was tested with Kolmogorov–Smirnov test and histograms.

One-way ANOVA was performed to analyse differences of GBA among flexion grades (0, 30, 60, 90, 120°). Multiple post-hoc testing was Bonferroni corrected.

The effect of distalisation, anteriorisation of the FTE and knee flexion on GBA, GBA-excursion and tunnel length were analysed using a linear regression model. Linearity and absent collinearity of the variables and respective errors were confirmed using scatterplots and histograms. To assess a potential change of effect size in a functional ROM close to extension (0, 30, 60°) solely, the linear regression model was repeated for this subgroup in an analogue manner. Effect sizes were calculated as Cohen’s D (f^2^) and graded as weak: < 0.02, medium: 0.15–0.35 and strong effect > 0.35 [9].

## Results

**Fig. 5:**
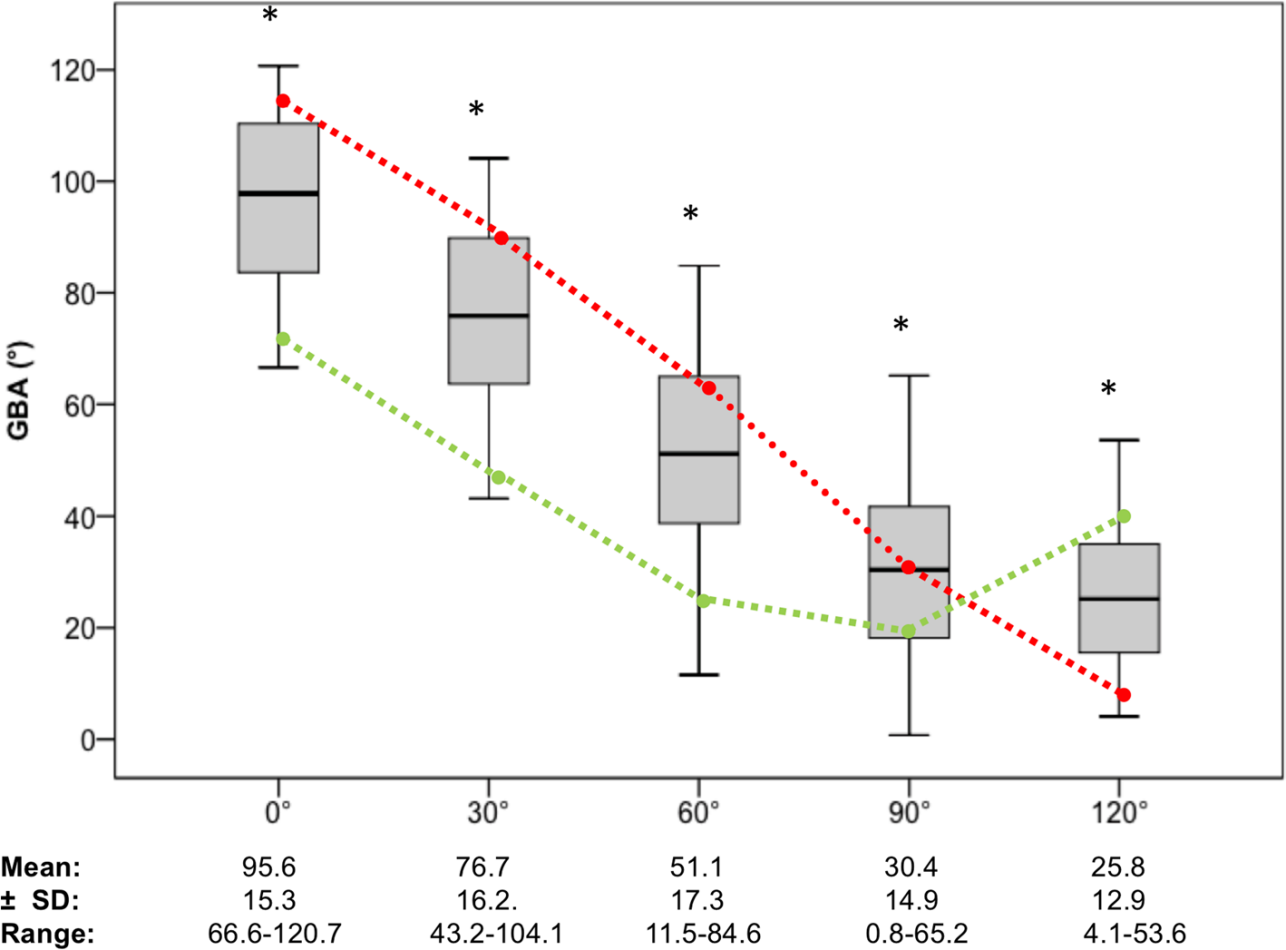
Mean graft bending angle (GBA) for all subjects and FTE among flexion grades. GBA: Graft bending angle. SD: Standard deviation. Boxplots depicts mean (line), 1st and 3rd quartile (box), minimum and maximum (whisker). Significant differences between groups after Bonferroni correction marked with * (90° vs. 120° *p *= 0.002), remaining: *p *< 0.001; ANOVA). Red dotted line: FTE with the greatest mean GBA-excursion; ΔGBA = 102.1 ± 6.3° (most distal-anterior FTE). Green dotted line: FTE with the smallest mean GBA-excursion; ΔGBA = 21.7 ± 6.9° (most proximal-posterior FTE)

Overall, the mean GBA was highest in 0° flexion and lowest in 120° (*p *< 0.001); (90° vs. 120° *p *= 0.002, remaining differences between flexion grades: *p *< 0.001) (Fig. 5).

The linear regression model was highly significant; F(3,399) = 638,22, *p *< 0.001, n = 400 and 82.9% of variance is explained by the FTE distalisation, anteriorisation and knee flexion, which corresponds to a strong effect (f^2^ = 4.85). A more distal location of the FTE along the Blumensaat’s line resulted in an increase of the GBA by 8.5 ± 0.6° /cm (*p *< 0.001). A more anterior FTE resulted in a decrease of GBA by -2.3 ± 0.6°/ cm (*p *< 0.001). Knee flexion led to a decrease of GBA by -18.6 ± 0.5° /30° of knee flexion (*p *< 0.001).

The repeated linear model close to extension (0–60°) showed an increase in effect size; F(3,239) = 777.32, *p *< 0.001, n = 240. 90.7% of variance is explained by the previous described three factors, which corresponds to a strong effect (f^2^ = 9.75). A more distal location of the FTE along the Blumensaat’s line resulted in an increase of the GBA by 13.0 ± 0.4° /cm (*p *< 0.001). A more anterior FTE resulted in an increase of GBA by 0.6 ± 0.4°/cm (*p *< 0.001). Knee flexion led to a decrease of GBA by -22.3 ± 0.6° /30° of knee flexion (*p *< 0.001).

Both, a more distal and a more anterior location of the FTE along the Blumensaat’s line resulted in an increase of GBA-excursion by 17.6 ± 1.1° /cm (*p *< 0.001) and 9.8 ± 1.1 /cm (*p *< 0.001), respectively; (F(2,79) = 160.70, *p *< 0.001, n = 80; f^2^ = 4.05).

Mean tunnel length was 38.5 ± 5.2 mm (range 29.6–50.5). Both, a more distal and anterior location of the FTE along the Blumensaat’s line resulted in an increase of tunnel length *corr* by 1.1 ± 0.3 /cm (*p *< 0.001) and 3.6 ± 0.3 /cm (*p *< 0.001), respectively; F(2,79) = 110.33, *p *< 0.001, n = 80; (f^2^ = 2.77). Posterior tunnel blow-out did not occur for any FTE.

## Discussion

The most important finding of this study is that a more proximal and posterior FTE, with respect to Blumensaat’s line, reduces GBA and GBA-excursion while preserving adequate tunnel length. Overall, GBA is reduced with increasing knee flexion.

**Fig. 6:**
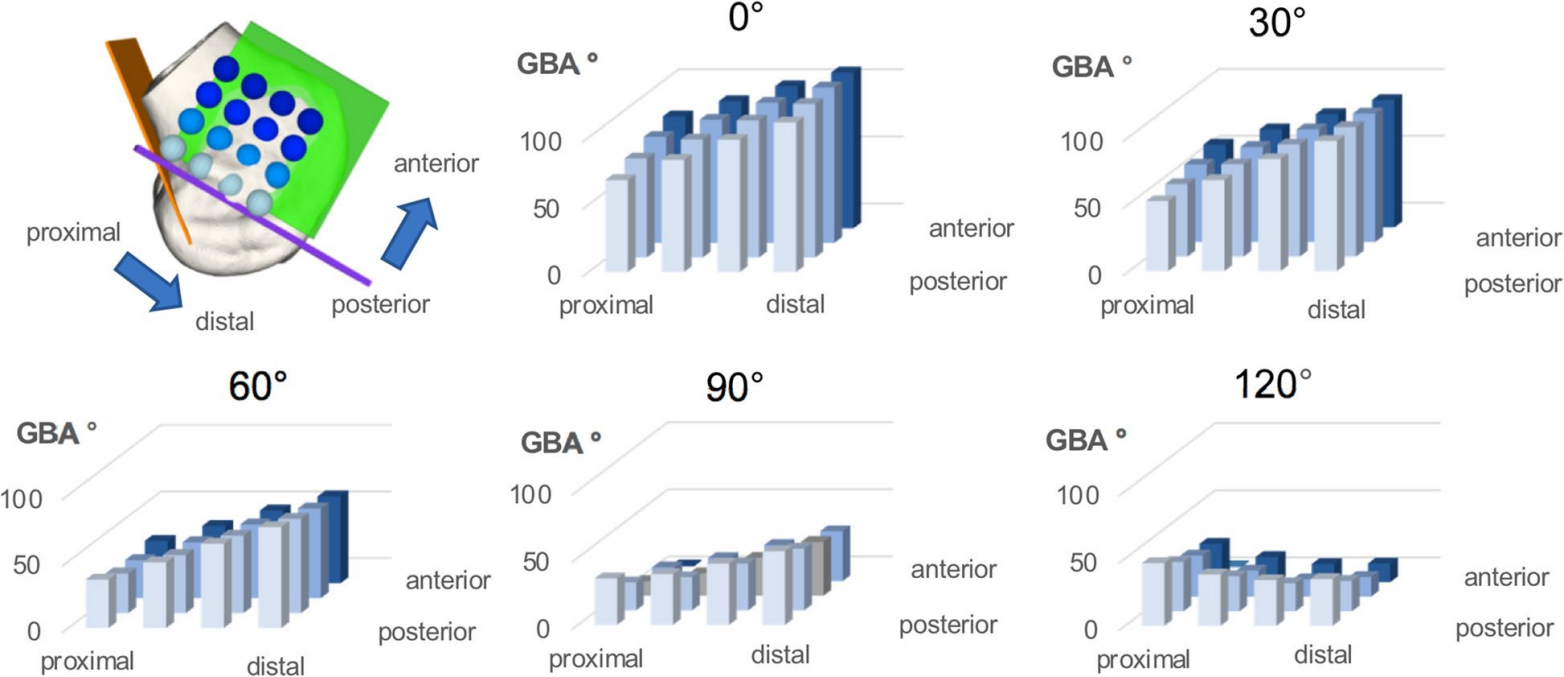
Mean GBA among flexion grades and femoral exit points. GBA: graft bending angle. 3D bar chart depicts GBA (y-axis) for each flexion° and for each FTE. All bar charts are oriented as in the model on the top left, listing the FTE with respect to the Blumensaat’s line (x-axis (rows; proximal to distal) and z-axis (lines; posterior to anterior)

The distalisation of the FTE showed the highest impact on GBA, while the anteriorisation had a considerably minor impact on GBA. Both, distalisation and anteriorisation, showed strict linearity until 60° of knee flexion but the effect of anteriorisation on GBA inverted in 90 and 120° and distalisation inverted in 120° of knee flexion (Fig. 6). This is also highlighted by the substantial increase of the effect size of the linear model for the subgroup close to extension (0–60°). However, mean GBA trebles in extension (0°) compared to flexion (90,120°) (Fig. 5) and despite the inverted relationship of the GBA in flexion compared to extension, the GBA for every FTE decreased throughout the full ROM. Therefore, these results strongly support to aim for the lowest GBA and GBA-excursion in full extension, which corresponds to the most posterior and proximal FTE. Additionally, most weight-bearing activities and rehabilitation exercises take place near full knee extension, which supports its clinical importance [4]. Looking at the GBA-excursion reveals the analogue behaviour as described for the GBA and underlines the relevance of a proximal and posterior FTE.

Finally, an adequate length for all femoral tunnels was preserved for all simulations if FTE is placed above the Blumensaat’s line and tunnel blow-out did not occur if the described landmarks (Blumensaat’s line and posterior cortical tangent) were respected [28].

Looking at the most proximal posterior FTE vs. most distal anterior demonstrates a halving of GBA and a five-fold decrease of GBA-excursion but the clinical relevance of this finding remains debatable. The role of GBA has been studied previously in regard to tunnel widening [33] and graft maturation [7, 17, 27]. Moreover, in posterior cruciate ligament (PCL) reconstruction a sharp GBA has been shown to not only attenuate the graft due to repetitive friction between the graft and tunnel inlet but even leading to graft displacement and residual laxity [18]. To decrease friction and graft strain, chamfering the intra-articular femoral tunnel inlet poses a surgical option. Graft maturation and bone tendon healing at femoral tunnel aperture are crucial issues that have not been resolved in ACL reconstruction so far and potentially delay rehabilitation, return to sports and ultimately may increase the risk of graft failure [8, 22].

The clinical applicability of our findings especially aids surgeons who perform an outside-in technique as they can define the FTE (respectively starting point) arbitrarily [19, 23], or if intraoperative fluoroscopy is used as in all-epiphyseal ACL reconstruction techniques in a paediatric population, for example [15]. However, aiming for the proposed optimal FTE position (posterior, proximal) in all-epiphyseal techniques is strictly limited by the epiphyseal line that only allows for a more distal FTE and a higher GBA respectively. Another important aspect that will allow the guidance of the FTE is the potential for navigated tunnel placement, for example, using augmented-reality [12, 26].

A limitation of the study is the relatively small sample size of five legs that did not allow the analysis of intra-subject morphometric anatomic variants. However, 16 FTE have been simulated in one-centimeter steps throughout the full ROM, resulting in 400 simulations. We are aware of the inverted relationship between FTE and GBA and GBA-excursion during deep knee flexion, which has been addressed by a subgroup analysis near extension (0–60°) and resulted in a very strong effect size of the model. The impact of in vivo kinematics on GBA in various activities can not be drawn from this study design. However, the use of a weight-bearing model revealed consistent outcomes compared to previous in-vivo results [31]. The impact of GBA on clinical outcome and the intraoperative applicability to guide the FTE remain subjects of further research.

## Conclusion

Aiming for a more proximal and posterior FTE, with respect to Blumensaat’s line, reliably reduces GBA and GBA-excursion, while preserving adequate tunnel length. This might aid to reduce excessive graft stress at the femoral tunnel aperture, femoral tunnel widening and enhance graft-healing.

## Abbreviations

3D: Three-dimensional
ACL: Anterior cruciate ligament
CT: Computer-tomography
FTE: Femoral tunnel exit
GBA: Graft bending angle
MRI: Magnetic resonance imaging
ROM: Range of motion
SNQ: Signal/noise quotient
